# Nurses’ experiences of the causes of their lack of interest in working in psychiatric wards: a qualitative study

**DOI:** 10.1186/s12912-021-00766-1

**Published:** 2021-12-09

**Authors:** Narges Rahmani, Eesa Mohammadi, Masoud Fallahi-Khoshknab

**Affiliations:** 1 Department of Nursing, Comprehensive health Research Center, Babol Branch, Islamic Azad University, Babol, Iran; 2 Nursing Department, Medical Sciences Faculty, Tarbiat Modares University, Nasr Bridge, Jalal Al-e Ahmad, Tehran, Iran; 3 Nursing Department, Medical Sciences Faculty, University of Social Welfare and Rehabilitation Sciences, Tehran, Iran

**Keywords:** Psychiatric nurse, Qualitative study, Interest

## Abstract

**Background:**

The shortage of psychiatric nurses is a major healthcare challenge. Lack of interest (LOI) contributes to the shortage of psychiatric nurses. Nonetheless, there are limited studies in this area. The present study was conducted to explore nurses’ experiences of the causes of their LOI in working in psychiatric wards.

**Methods:**

This qualitative study was conducted in 2016–2019 using the content analysis approach. Participants were 27 nurses purposively recruited with maximum variation from the psychiatric wards of three referral hospitals in Iran. Data were collected via unstructured interviews and were concurrently analyzed using the conventional content analysis approach recommended by Graneheim and Lundman.

**Results:**

The causes of participants’ LOI in working in psychiatric wards were grouped into three main categories, namely inadequate professional skills for psychiatric care practice, negative public attitude towards psychiatric nurses, and concerns over patients.

**Conclusion:**

This study suggests that the causes of nurses’ LOI in working in psychiatric wards are not only personal, but also social and organizational. Findings help managers and authorities develop strategies to increase psychiatric nurses’ interest in working in psychiatric wards through improving their work conditions and professional knowledge and skills.

## Background

With high prevalence and heavy disease burden, psychiatric disorders are among the major healthcare challenges [1]. According to the World Health Organization report, 450 million people in the world suffer from psychiatric disorders, mainly major depression [2]. This organization also reports that 25% of people suffer from one or more psychiatric disorders in each stage of life [3]. A study in Iran also showed that in 2003, mental and behavioral disorders were responsible for 16% of total disease burden and were the second highest ranked disorders with respect to disease burden [1].

Patients with psychiatric disorders are at high risk for death due to suffering from different comorbid physical conditions, comorbid cognitive impairments, refusal of treatment, and healthcare providers’ biases [4]. Moreover, these patients need to use multiple medications, are at risk for medication side effects, have unhealthy lifestyle, are overweight or obese, use tobacco, and hence, need quality healthcare services [5]. Quality healthcare delivery in turn depends on the availability of competent and committed healthcare providers, including nurses.

Nurses are the largest group of healthcare providers in psychiatric hospitals. The main goals of nursing care in these hospitals are to provide quality care to patients in order to facilitate their recovery and help them return to normal life. Consequently, quality nursing care services can help patients in psychiatric hospitals more rapidly return to normal life and prevent their re-hospitalization [6]. Nonetheless, there are insufficient nurses for psychiatric care delivery. The World Health Organization reported that in 2014, there were less than one psychiatrist and 7.7 nurses per 100,000 patients in psychiatric settings, implying severe shortage of psychiatric care providers [2].

Nurses’ lack of interest (LOI) in working in psychiatric settings is a major factor contributing to the shortage of nursing staff in these settings. A study reported that most nurses choose to work in psychiatric settings only when there is no other employment opportunity [7]. Factors affecting their interest in working in psychiatric settings are their negative attitudes towards work in these settings [8], stress, and emotional burnout [9]. A study reported that more than half of the nurses in psychiatric settings suffer from severe stress and emotional burnout [9]. Moreover, a study showed that nursing students are not willing to work in psychiatric settings due to different stressors associated with work in these settings, emotional burnout, and negative public attitudes towards psychiatric settings [10]. Other causes for nurses’ LOI in working in psychiatric settings include lack of technical nursing practice in these settings [11] and stressful work conditions [12].

A review of studies conducted in this regard showed some studies have identified sources and effects of carer fatigue and burnout for mental health nurses [10] or only examined the attitude of nurses towards people with mental illness [12]. Some also examined the factors influencing students’ interest [11, 13]. Since qualitative studies are context-based and different results are obtained in different fields. However, previous studies have not specifically explored nurses’ experiences of the causes of their LOI in working in psychiatric wards. This consideration coupled with the considerable impact this LOI has on the shortage of psychiatric nurses and the quality of psychiatric nursing care [5, 14, 15] has led to the formulation of the present research question. The aim of this qualitative study was to explore nurses’ experiences of their LOI in working in psychiatric wards.

## Methods

### Design

This qualitative study was conducted in 2016–2019 using the content analysis approach. Qualitative research provides the opportunity to explore experiences in the natural environment. The choice of method depends on the aim of the study [16]. The aim of this study was to explore nurses’ experiences of the causes of their LOI in working in psychiatric wards. When there is not enough knowledge about a phenomenon or existing knowledge is not integrated, the inductive content analysis approach is recommended [16]. According to Wildemuth (2009) Content analysis is a research method for making replicable and valid inferences from data to their context, with the purpose of providing knowledge, new insights, a representation of facts, and a practical guide to action [17]. A genuinely qualitative content analysis approach, as recommended by Graneheim and Lundman [18] was employed.

Thus, considering that there was not enough knowledge about the experiences of the causes of LOI in working in psychiatric wards in nurses in the mentioned context and to achieve the research goal, a qualitative manifest content analysis method was used.

### Setting & sample

Study setting was the psychiatric wards of two referral hospitals in Mazandarn province in the north of Iran and a referral psychiatric hospital in Tehran, Iran [19]. Participants were 27 hospital nurses purposively recruited to the study. Inclusion criteria were ability to communicate verbally and working in psychiatric ward. The research samples were nurses with bachelor’s or master’s degrees who worked in psychiatric wards. All participants completed 4 theoretical units and 2 mental health internship units at the university. Sampling was done with maximum variation concerning eligible participants’ gender, educational level, work experience, and affiliated ward to provide rich, wide, and deep information to gain possible maximum variation in sampling. Selection of variables was informed by the literature [20].

### Data collection procedures

Data were generated by unstructured interviews which were started using a broad open-ended question, i.e. “May you please talk about you interest in working in this ward?”. Then, clarifying questions were used based on participants’ responses and the research goals. Examples of these questions were “What contributed to your lack of interest?”, “What happened next?”, “Can you please explain more?” “What do you mean by this?”. The first author conducted the interviews after introducing himself and the objectives of the study.

Interviews were conducted at the request of the participants at the participants’workplace.

Data collection continued until the development of categories and data saturation. The length of the interviews was 32 min, on average. All interviews were held in a quiet room in participants’ workplace and were recorded with their consent.

### Data analysis

The conventional content analysis approach recommended by Graneheim and Lundman was used for data analysis [18]. The first author primarily listened to each interview for several times and transcribed it in order to get familiar with the data. Then, meaning units were identified and coded and the codes were grouped into more abstract subcategories according to their similarities. Subcategories were also compared with each other and grouped into main categories. In a manifest analysis, the researcher describes what the informants actually say, stays very close to the text, uses the words themselves, and describes the visible and obvious in the text. Therefore, manifest content analysis was used in the present study [21, 22].

Table 1 shows a sample of data analysis.

**Table 1:** An example of data analysis

Meaning units	Primary codes	Subcategories	Categories
I feared patients with schizophrenia, specifically those who were tall and giant. They look at you in a different way (P. 23). We are not secure in this ward. Occupational stress and fear are very great. Of course, outsiders may not feel so (P. 18).	Fear over patients with schizophrenia Occupational stress due to feeling insecure	Fear over patient assault	Concerns over patients
In the first days, I had fear over patient assault due to my limited knowledge about them and their conditions (P. 21). I had not adequately experienced psychiatric wards during my studentship. Hence, I felt great stress in the first days of my work in this ward even when I wanted to establish effective communication with patients (P. 20).	Fear over being injured by patients due to having little knowledge about them, their conditions, and how to establish effective communication with them	Fear due to unfamiliarity with patients and their conditions	

### Trustworthiness

Trustworthiness was established via the four criteria of credibility, dependability, confirmability, and transferability [19]. To ensure credibility, the first author had direct communication with participants over twelve months. The researcher tried to be adequately sensitive to the participants with prolonged engagement; For the convergence of data with experience, the primary codes were checked by five participants and made corrections wherever necessary. To ensure the confirmability of the data, two qualitative researchers checked the codes and the categories that emerged from the interviews. The researcher obtained the data and performed the interviews by asking the later probing questions based on the participants’ previous answers without personal bias. Also, in the data analysis, she extracted the codes without personal bias. To ensure the dependability of the study results, methods utilized for coding concepts and themes, as well as textual and audio data became available, Furthermore, two of the authors assessed and commented on the generated codes and categories. Sampling with maximum variation concerning participants’ gender, educational level, and affiliated ward also helped ensure transferability. There were also attempts to select the study samples completely based on the objectives of the study and without any bias.

### Ethical considerations

The Ethics Committee of Tarbiat Modares University, Tehran, Iran, approved this study (code: IR.TMU.REC.1394.169). Participants received clear information about the aim of the study and interviews were held according to their preference. They were ensured about their freedom to voluntarily withdraw from the study, the confidentiality of their data, and their access to the study findings at will. Necessary permissions for the study were also obtained from the authorities of the study setting.

## Results

Study participants were 27 nurses from three referral hospitals in Iran. Most participants were female (Sixteen participants), had bachelor’s degree (21 participants), and worked in acute care wards (twenty participants). All demographic characteristics of the participants are presented in Table 2.

**Table 2:** The demographic characteristics of The nurses who worked in psychiatric wards

Characteristics	Values
Gender	
Male	11
Female	16
Education	
Bachelor’s Degree	21
Master’s Degree	6
Ward	
Acute	20
Chronic	5
Pediatric	2
Hospital	
Zare Sari	12
babol	5
Razi tehran	10
Years of Service	
≤ 2	9
3–8	8
8–14	3
14–20	3
≥ 20	4

Participants’ experiences of the causes of their LOI in working in psychiatric wards were grouped into eight subcategories and three main categories, namely inadequate professional skills for psychiatric care practice, negative public attitudes towards psychiatric nurses, and concerns over patients (Table 3).

**Table 3:** The subcategories and categories of the study

Subcategories	Categories
Forced choice of working in psychiatric wards	Inadequate professional skills for psychiatric care practice
Inappropriate work conditions	
Lack of the required professional knowledge for working in psychiatric wards	
Colleagues’ negative attitudes towards psychiatric nurses	Negative public attitude towards psychiatric nurses
Families’ negative attitudes towards psychiatric nurses	
Negative experiences of psychiatric nursing from their studentship period	
Fear over patient assault	Fear of patients
Fear due to unfamiliarity with patients and their conditions	

### Inadequate professional skills for psychiatric care practice

Inadequate professional skills for psychiatric care practice was one of the main causes of LOI among participants. Participants noted that they were not ready enough for psychiatric care practice when starting their work in psychiatric wards and had chosen this work because they had no more option. They considered work in psychiatric wards to be harder than work in other hospital wards due to factors such as insignificant patient recovery, limited readiness for work, and finding no pleasure at work. The subcategories of this category were forced choice of working in psychiatric wards, inappropriate work conditions, and lack of the necessary professional knowledge for working in psychiatric wards.

#### Forced choice of working in psychiatric wards

Most participants reported that they were compelled to work in psychiatric wards due to their own physical problems, heavy workload in other wards, or nursing staff shortage in psychiatric wards. Some of them also noted that they chose to work in psychiatric wards due to advantages such as reducedor less working hours, earlier retirement, no night shift, and more leaves.

*I had undergone a surgery and my doctor had emphasized that I shouldn’t work in infectious diseases wards. Therefore, I chose psychiatric care practice, stayed in it, and couldn’t change it. I chose it without any interest (P. 19; a male nurse with a ten-year work experience).*

#### Inappropriate work conditions

Participants reported that they had limited interest in working in psychiatric wards due to repetitious nature of work in these wards, limited patient recovery, patients’ frequent re-hospitalizations, their own limited readiness for psychiatric care practice, and subsequent considerable occupational strain. Participants report difficulty working in the psychiatric ward due to the patient’s lack of recovery, lack of noticeable change over time, and a pleasant feeling of nursing following treatment.

*Psychiatric patients never get better. We do not have the pleasant feeling of nursing here, the feeling that the patient is going well and has gained a sense of well-being. (P.16; a female nurse with a seven-year work experience).*

#### Lack of the necessary professional knowledge for working in psychiatric wards

Participants reported that nurses in psychiatric wards have lower levels of professional knowledge compared with nurses in other hospital wards. They also referred to wide theory-practice gap in psychiatric wards and described these wards as wards with repetitious patients and limited range of diagnoses. Accordingly, they noted that they had limited interest in work, had fallen behind their colleagues in other wards with respect to professional competence, and had been fossilized.

*I didn’t have any interest in working in this ward because working in this ward lowers your professional level and knowledge. Repetitive tasks in these wards have resulted in rote learning and routine practice. When you stop doing these routine tasks for a while, you easily forget them. I know that I have fallen behind my peers in other wards (P. 14; a female nurse with a seventeen-year work experience).*

### Negative public attitudes towards psychiatric nurses

Negative public attitudes towards psychiatric nurses were another main cause of LOI in working in psychiatric wards. Participants noted that people, including their family members and relatives, have misconceptions and negative images about working in psychiatric hospitals. Hence, psychiatric nurses feel compelled to hide the name of their workplace from others despite several years of work experience in psychiatric wards. They reported family members’ and colleagues’ negative attitudes towards psychiatric nursing and their own past experiences during their university education as causes of their LOI in working in psychiatric wards. The subcategories of this category were colleagues’ negative attitudes towards psychiatric nurses, families’ negative attitudes towards psychiatric nurses, and negative experiences of psychiatric nursing from their studentship period.

#### Colleagues’ negative attitudes towards psychiatric nurses

Participants reported that their colleagues in other hospital wards had negative attitudes towards psychiatric nurses, asked them to change their workplace, and had asked them not to choose working in psychiatric settings at the time of employment. These factors had negatively affected their interest in working in psychiatric wards.

*Previously, I was in another hospital. When I successfully passed the employment exam, a nurse asked me whether I could change my workplace. It greatly disappointed me (P. 24; a female nurse with a fourteen-year work experience).*

#### Families’ negative attitudes towards psychiatric nurses

Participants noted that their family members and significant others had negative attitudes towards psychiatric nurses and did not have accurate understanding about psychiatric hospitals and patients. Therefore, our participants attempted to hide the name of their workplace from others. Family members’ negative attitudes had negatively affected their mood, caused them preoccupation with their work, and reduced their interest for work. They reported that their significant others considered psychiatric hospitals as a place for keeping patients rather than as medical centers and believed that patients in these centers were often restrained.

*I had no interest for working in psychiatric ward. I feel whatever I explain to my significant others, they don’t understand and just say that I work in madhouse (P. 17; a female nurse with a two-year work experience).*

#### Negative experiences of psychiatric nursing from their studentship period

Participants noted that they had been exposed to negative experiences and images of psychiatric nursing from their studentship period and hence, were not interested in working in psychiatric wards. Some of them reported that they had experienced patient assault during their studentship in psychiatric wards.

*I didn’t like psychiatric wards from my studentship period because studentship in these wards had greatly affected me. Those days, I thought to myself that I would never choose working in psychiatric wards (P. 21; a female nurse with a six-year work experience).*

### Fear of patients

Fear over patient assault and unfamiliarity with patients were also among the causes of participants’ LOI in working in psychiatric wards. They noted that due to the negative attitudes of their friends, colleagues, and significant others towards patients with psychiatric disorders, they were fearful when they heard the expression “patients with psychiatric disorders” at the time of starting their work in psychiatric wards, attempted not to get close to them, and had anxiety over working in psychiatric wards. The two subcategories of this category were fear over patient assault and fear due to unfamiliarity with patients and their conditions.

#### Fear over patient assault

Participants had fear over patient assault because they believed that patients with psychiatric disorders might become irritable and aggressive. They were mostly concerned with assault by patients with schizophrenia due to their delusions and hallucinations, patients with paranoia, and physically strong patients. Such fear was a major cause for their LOI in working in psychiatric wards.

*I didn’t like to work here due to my fear over patient irritability. Patients with psychiatric disorders may suddenly become irritable and aggressive and attack you. I had fear over injury to myself (P. 24; a female nurse with a fourteen-year work experience who worked in men’s psychiatric ward).*

#### Fear due to unfamiliarity with patients and their conditions

Participants expressed that they were not interested in working in psychiatric wards due to their unfamiliarity with patients with psychiatric disorders and their unique characteristics. They were not familiar with these patients and their symptoms and hence, felt fearful when witnessing conditions such as panic attacks. They noted that they did not know how to manage unfamiliar conditions and hence, experienced high levels of stress.

*In the first days, I had no knowledge about these patients and their problems and hence, I feared them and didn’t want to work in this ward (P. 21; a female nurse with a six-year work experience).*

## Discussion

Nurses’ lack of interest (LOI) in working in psychiatric settings is a major factor contributing to the shortage of nursing staff in these settings. Therefore, The present study was conducted to explore nurses’ experiences of the causes of their LOI in working in psychiatric wards. The findings suggest that nurses’ perception of causes of LOI in working in psychiatric wards were inadequate professional skills for psychiatric care practice, negative public attitudes towards psychiatric nurses, and Fear of patients.

One of the main causes of LOI in working in psychiatric wards was inadequate professional skills for psychiatric care practice. Participants noted that their forced choice of working in psychiatric wards, inappropriate work conditions, patients’ insignificant recovery, and lack of the necessary professional knowledge for working in psychiatric wards contributed to their LOI in working in psychiatric wards. In line with these findings, a former study reported that nurses who were employed for working in psychiatric wards did not take appropriate ward orientation courses and had to immediately start working due to staff shortage [20]. Another study showed that most psychiatric nurses would quit nursing during the first twelve months after graduation due to factors such as heavy workload, poorly defined career structure, and limited number of experienced physicians [23]. Moreover, a study reported that psychiatric nurses with closer professional relationships with patients had more positive attitudes towards working in psychiatric wards [12]. Education is a major factor affecting students’ and nurses’ interest in work. Therefore, providing them with quality education about skills and competencies for psychiatric nursing practice can increase their interest. Using experienced nurses for supervising and mentoring novice nurses can also increase nurses’ interest in working in psychiatric wards. A former study highlighted that improving nursing students’ theoretical and practical knowledge was effective in developing positive attitudes towards psychiatric nursing [24]. Contrastingly, a study found that the majority of students hold a positive attitude towards people with mental illness and are satisfied with their preparation in mental health nursing [25]. These findings are inconsistent with those of the present study and those of other research and this difference may be attributed to the specific context of the study.

The second main category of the causes of nurses’ LOI in working in psychiatric wards was negative public attitudes towards psychiatric nurses. Participants noted that their colleagues’ and families’ negative attitudes towards psychiatric nurses and their own negative past experiences of psychiatric nursing contributed to their LOI. Research result show that the lowly status of psychiatric nursing has been an issue for decades [26]. Moreover, numerous academics globally have studied the phenomenon of associative stigma [27, 28]. The low status of the psychiatric nursing profession may also be due to the fact that many people have misconceptions about the people who work with this population. This in turn reduces their interest in working in psychiatric wards [29]. Nurses may be influenced by these beliefs and may project their frustration on patients who eventually come to be perceived as less deserving of care and support, and reduce their interest in working in psychiatric wards [30]. Two former studies also reported that negative attitudes towards psychiatric nurses negatively affect nurses’ interest in working in psychiatric wards and their morale [23, 31]. Another study reported that fear over social stigmatization prevented psychiatric nurses from informing their friends and families about their workplace [20]. Moreover, significant others’ negative attitudes and beliefs about patients with psychiatric disorders and psychiatric nurses negatively affect nurses’ morale and cause them intense preoccupation with their work. These problems are mostly prevalent among psychiatric nurses, while nurses in other hospital wards may have positive feelings about their work, particularly at the time of employment [32–35]. To reduce association stigma, Bladon suggested that mental health nurses first need to identify their areas of uniqueness then celebrate this uniqueness through public means [36]. The unique characteristics were that psychiatric nurses use specialized knowledge in mental health, mental illness, and addictions as the central theme that influences the use of the therapeutic relationship, holistic approach, recovery, stigma reduction, and advocacy for system change. These unique characteristics could be used as the foundation for the promotion of the profession and the education of other health professionals [37].

Concerns over patients was the third main category of the study. Participants noted that their fear over patient assault and their unfamiliarity with psychiatric wards and patients significantly affected their interest in working in psychiatric wards. Consequently, they preferred to work in wards where they do not experience fear and anxiety. A former study in Iran also reported that novice psychiatric nurses were not interested in working in psychiatric wards due to their fear over unexpected events and affliction by psychiatric disorders [20]. Another study showed that psychiatric nurses had fear over communicating with patients and attending psychiatric wards [12]. Patients’ irritability and aggressiveness coupled with nurses’ perceived inadequacy in dealing with such patients results in fear and stress [33–35, 38].. Whilst nurses on other wards mostly fear committing errors or causing injuries to patients, participants in this study reported their greatest fears centered around patient assaults. Nursing students also fear attending psychiatric wards because they may believe that patients with psychiatric disorders are dangerous. Therefore, interventions are needed to develop positive attitudes about psychiatric wards among students. An example of such interventions is the provision of the opportunity for having clinical experiences [8].

Students should be exposed to mental health nursing through clinical experiences, and theory classes, in helping them make an informed decision in their eventual career choice [8]. inclusion of service-users during lectures in curricula to enhance continuum beliefs. This is an educational measure to target this fear.

The present study, by discovering nurses’ experiences of the reasons for the lack of interest in working in the psychiatric ward, can provide useful information to educational administrators to take an important step in improving the quality of care by increasing interest in work.

Given that the participants were university graduates where the researcher worked as an instructor, this could have prevented participants from sharing all of the participants’ experiences during the interview. However, the researcher tried to overcome this limitation by sampling different hospitals and nurses. But there were some limitations that prevented participants from achieving all aspects of their experiences. Achievement to more diverse participants were limitations of the present study that may affect the transferability of the result. The views of other general nurses were not explored to triangulate the findings in this study.

## Conclusion

This study shows that the major causes of nurses’ LOI in working in psychiatric wards are their inadequate professional skills for psychiatric care practice, negative public attitudes towards psychiatric nurses, and concerns over patients with psychiatric disorders. Nurses in psychiatric wards lack knowledge and familiarity regarding psychiatric care practice and psychiatric wards and choose working in these wards when they have no more options. Their interest in working in psychiatric wards is also affected by their families’ and colleagues’ attitudes towards psychiatric nursing, their own past experiences in this area, and their fear and anxiety over patient assault.

The findings can help nursing managers and policymakers know the nurses’ causes of LOI in working in psychiatric wards and to make new changes for their preparation and improve the quality of care. They can design and implement a workshop or codified program focusing on clinical supervision and a culture of reflective practice to strengthen nurses’ awareness in dealing with patients. They also allow nurses to attend courses/ training sessions during work hours to create a culture of continuous learning. Media-based interventions are also needed to modify public attitudes towards patients with psychiatric disorders and their healthcare providers. Such interventions may increase nurses’ interest in working in psychiatric wards and improve the quality of their care services.

The results of the present study can complement the quantitative studies conducted in this regard since it uncovered the nurses’ lived experiences of causes of LOI in working in psychiatric wards. Since this study highlighted the different aspects and elements of causes of nurses’ LOI in working in psychiatric wards, it can be used to develop a tool to measure nurses’ perceptions in this field. Therefore, studies on tool design of psychiatric nurses’ perceptions of causes of LOI or Mixed Methods are recommended. Also a study with other general nurses in a different field is recommended.

## Data Availability

The datasets generated and/or analysed during the current study are not publicly available due [individual privacy could be compromised] but are available from the corresponding author on reasonable request.

## Abbreviations

LOI: Lack of interest
